# Clinical applicability of quantitative atrophy measures on MRI in patients suspected of Alzheimer’s disease

**DOI:** 10.1007/s00330-021-08503-7

**Published:** 2022-05-31

**Authors:** Silvia Ingala, Ingrid S. van Maurik, Daniele Altomare, Raphael Wurm, Ellen Dicks, Ronald A. van Schijndel, Marissa Zwan, Femke Bouwman, Niki Schoonenboom, Leo Boelaarts, Gerwin Roks, Rob van Marum, Barbera van Harten, Inge van Uden, Jules Claus, Viktor Wottschel, Hugo Vrenken, Mike P. Wattjes, Wiesje M. van der Flier, Frederik Barkhof

**Affiliations:** 1 Department of Radiology and Nuclear Medicine, Amsterdam Neuroscience, Vrije Universiteit Amsterdam, Amsterdam University Medical Center, Location VUmc, PO Box 7057, 1007 MB Amsterdam, The Netherlands; 2 Department of Radiology and Nuclear Medicine, Noordwest Hospital Group, Alkmaar, The Netherlands; 3 Alzheimer Center Amsterdam, Department of Neurology, Amsterdam Neuroscience, Vrije Universiteit Amsterdam, Amsterdam UMC, Location VUmc, Amsterdam, The Netherlands; 4 Department of Epidemiology and Data Science, Amsterdam UMC, Location VUmc, Amsterdam, The Netherlands; 5 Laboratory of Neuroimaging of Aging (LANVIE), University of Geneva, Geneva, Switzerland; 6 Memory Clinic, University Hospitals of Geneva, Geneva, Switzerland; 7 Department of Neurology, Medical University of Vienna, Vienna, Austria; 8 Geriatric Department, Noordwest Ziekenhuis Groep, Alkmaar, The Netherlands; 9 Geriatric Department, Noordwest Ziekenhuis Groep, Alkmaar, The Netherlands; 10 Department of Neurology, Elisabeth-TweeSteden Ziekenhuis, Tilburg, The Netherlands; 11 Department of Geriatrics, Jeroen Bosch Hospital, ‘S-Hertogenbosch, The Netherlands; 12 Department of Family Medicine and Elderly Care Medicine, Vrije Universiteit Amsterdam, Amsterdam UMC, Amsterdam, The Netherlands; 13 Department of Neurology, Medisch Centrum Leeuwarden, Leeuwarden, The Netherlands; 14 Department of Neurology, Catharina Hospital, Eindhoven, The Netherlands; 15 Department of Neurology, Tergooi Hospital, Blaricum, The Netherlands; 16 Department of Diagnostic and Interventional Neuroradiology, Hannover Medical School, Hannover, Germany; 17 Institutes of Neurology and Healthcare Engineering, UCL, London, UK

**Keywords:** Alzheimer’s disease, Magnetic resonance imaging (MRI), Hippocampal volume (HCV), Gray matter volume (GMV), Visual rating scales

## Abstract

**Objectives:**

Neurodegeneration in suspected Alzheimer’s disease can be determined using visual rating or quantitative volumetric assessments. We examined the feasibility of volumetric measurements of gray matter (GMV) and hippocampal volume (HCV) and compared their diagnostic performance with visual rating scales in academic and non-academic memory clinics.

**Materials and methods:**

We included 231 patients attending local memory clinics (LMC) in the Netherlands and 501 of the academic Amsterdam Dementia Cohort (ADC). MRI scans were acquired using local protocols, including a T1-weighted sequence. Quantification of GMV and HCV was performed using FSL and FreeSurfer. Medial temporal atrophy and global atrophy were assessed with visual rating scales. ROC curves were derived to determine which measure discriminated best between cognitively normal (CN), mild cognitive impairment (MCI), and Alzheimer’s dementia (AD).

**Results:**

Patients attending LMC (age 70.9 ± 8.9 years; 47% females; 19% CN; 34% MCI; 47% AD) were older, had more cerebrovascular pathology, and had lower GMV and HCV compared to those of the ADC (age 64.9 ± 8.2 years; 42% females; 35% CN, 43% MCI, 22% AD). While visual ratings were feasible in > 95% of scans in both cohorts, quantification was achieved in 94–98% of ADC, but only 68–85% of LMC scans, depending on the software. Visual ratings and volumetric outcomes performed similarly in discriminating CN vs AD in both cohorts.

**Conclusion:**

In clinical settings, quantification of GM and hippocampal atrophy currently fails in up to one-third of scans, probably due to lack of standardized acquisition protocols. Diagnostic accuracy is similar for volumetric measures and visual rating scales, making the latter suited for clinical practice.

**Summary statement:**

In a real-life clinical setting, volumetric assessment of MRI scans in dementia patients may require acquisition protocol optimization and does not outperform visual rating scales.

**Key Points:**

• *In a real-life clinical setting, the diagnostic performance of visual rating scales is similar to that of automatic volumetric quantification and may be sufficient to distinguish Alzheimer’s disease groups*.

• *Volumetric assessment of gray matter and hippocampal volumes from MRI scans of patients attending non-academic memory clinics fails in up to 32% of cases*.

• *Clinical MR acquisition protocols should be optimized to improve the output of quantitative software for segmentation of Alzheimer’s disease–specific outcomes*.

**Supplementary Information:**

The online version contains supplementary material available at 10.1007/s00330-021-08503-7.

## Introduction

Alzheimer’s dementia (AD) is the final clinical stage of Alzheimer’s disease, a progressive neurodegenerative condition leading to neuronal loss [1]. MRI is recommended at least once in the diagnostic workup of patients attending memory clinics, as it improves diagnostic sensitivity and specificity when used in combination with other biomarkers [2–4]. Structural brain MRI provides a non-invasive and reliable way of quantitively assessing the degree of atrophy in vivo through measures of global and regional volumes that have proven valuable in identifying subjects at risk of cognitive decline even before the occurrence of dementia [1, 5]. The latest clinical and research guidelines for the definition of Alzheimer’s disease recommend to include MRI in the assessment of potentially at-risk individuals and quantify neurodegeneration [6–10].

Assessment of MRI scans in the clinical setting relies mostly on the detection of patterns of generalized or medial temporal, parietal, and global cortical atrophy in the brain [5, 11], often supported by visual rating scales [1, 2, 12]. Methods for quantification of (regional) atrophy have so far been mostly restricted to the research domain. In real-life clinical settings, successful efforts to go beyond descriptive radiological reports both in academic and non-academic centers have been reported, but the widespread use of quantification methods is still hampered by lack of neuroradiologists’ training, lack of requests by the clinicians, and time issues [13].

While visual inspection using rating scales is not very demanding, this method has some degree of subjectivity and it is dependent on the rater’s experience. Conversely, quantitative methods may provide more objective and sensitive readouts, but are more time-consuming and their output might be affected by the quality of the scans [12, 14–16]. While many methods for quantification are available for research purposes, their value in the clinical setting has not been investigated yet, and they require a higher degree of standardization, being sensitive to MRI acquisition parameters [17].

We aimed to use clinical MRI scans from a mono-center, academic (retrospectively acquired) and multi-center, non-academic (prospectively collected) memory clinics within The Netherlands to establish the feasibility of quantifying atrophy in real-life clinical settings and determine whether these techniques better distinguish diagnostic groups than visual rating scales. To this end, total gray matter volume (GMV) and hippocampal volume (HCV) were quantified with two different automated pipelines and the degree of atrophy was also assessed through visual rating scales. Quality control of routinely acquired scans and the output of quantitative pipelines were performed to establish whether clinical MRI scans are suitable for such measurements. Finally, we established whether quantitative and visual measures differed in diagnostic performance in both academic and non-academic real-life clinical settings.

## Materials and methods

### Study participants

This study used data acquired as part of the Alzheimer’s biomarkers in daily practice (ABIDE) project that focuses on the translation of knowledge on diagnostics test, including MRI, to daily clinical practice [18]. A total of 231 MRI scans from patients attending one of eight non-academic, local memory clinics (LMC) in The Netherlands [18] were prospectively collected between May 2015 and January 2017. Inclusion criteria were a Mini-Mental State Examination (MMSE) score ≥ 18 and the possibility of undergoing an MRI scan.

On the basis of clinical assessment, MRI, and performance in the neuropsychological assessment, subjects were classified as either cognitively normal (CN), with mild cognitive impairment (MCI), or with AD according to clinical criteria [9]. All subjects with a diagnosis of dementia other than AD were excluded from the study (*n* = 25).

The sample complemented with 492 patients retrospectively collected from Amsterdam Dementia Cohort (ADC) at the Amsterdam University Medical Center (UMC), location VUmc, with matching eligibility criteria [19], leading to a total of 698 subjects (LMC *n* = 206, ADC *n* = 492). All patients in ADC underwent a standardized clinical assessment including medical history, physical and neurological examination, laboratory tests, lumbar puncture, neuropsychological testing, and brain MRI. Clinical diagnoses were performed by a multidisciplinary team according to international guidelines [6–10].

All patients signed informed consent and the study was approved by the institutional ethical committee.

### MRI data acquisition and analyses

As a part of the routine clinical visit, anatomical T1-weighted (T1w) images were acquired on clinical MRI scanners with a field strength of either 1.5 T or 3 T using a spoiled gradient-echo type of sequence (e.g., MPRAGE, FSPGR, TFE). Depending on the acquisition site, the MRI protocol also included additional sequences to visually assess vascular pathology, exclude incidental findings, and help in establishing the clinical diagnosis.

Visual reads of the complete imaging dataset were performed by an experienced neuroradiologist (M.P.W.) blinded to clinical information. Visual reads were performed in native space using established, validated semiquantitative visual rating scales (medial temporal lobe atrophy scores, MTA 0–4; posterior cortical atrophy scores, PCA 0–3; global cortical atrophy scores, GCA 0–3, Fazekas score for white matter hyperintensities of probable vascular origin, 0–3) [12, 20, 21].

For volumetric outcomes, we selected two automated, model-based approaches for segmenting T1w images, FSL (v6.0, http://www.fmrib.ox.ac.uk/fsl/) and FreeSurfer v6.0, both easy to use, well documented, and freely available. Outcomes of interest were total GMV and HCV.

Using the FSL pipeline, GMV was derived, together with a scaling factor normalizing for brain size, via structural image evaluation using normalization of atrophy (SIENAX) [22]. Similarly, HCV was calculated using FIRST [23]. Left HCV and right HCV were averaged. Both GMV and HCV were normalized for brain size using the scaling factor.

Automated cortical parcellations in FreeSurfer were run using a default script template (recon-all). FreeSurfer image analysis suite performs cortical reconstruction and volumetric segmentation of T1w images into GM, white matter, and cerebrospinal fluid [24]. Left HCV and right HCV were averaged. Normalization of FreeSurfer-derived results was performed by correcting for the mean estimated total intracranial volume.

All scans were centrally collected in the Amsterdam University Medical Center (UMC) and analyses were performed by a single operator (S.I.) blinded to clinical information using identical pipelines. The output of FSL and FreeSurfer was visually inspected for image and segmentation quality by two experienced readers blinded to clinical information (S.I. and R.W.). Scans failed QC if at least one of the following occurred: lack of the appropriate sequence for analysis, incorrect registration or segmentation, failure of the pipeline, or implausible volume estimation.

### Statistical analyses

**Fig. 1:**
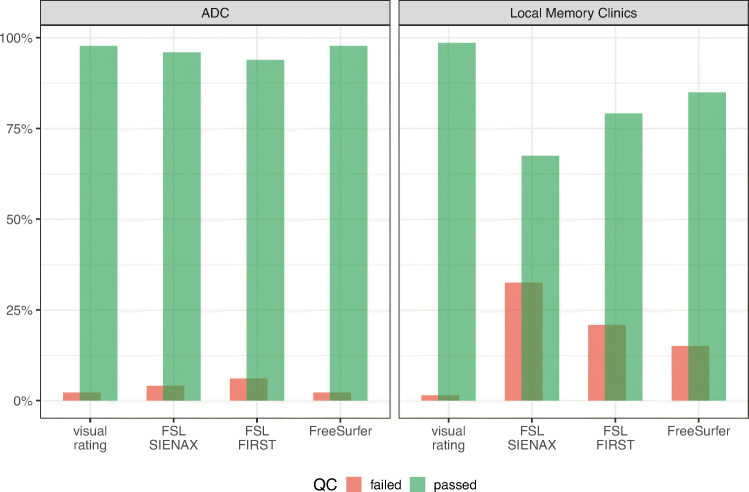
Overview of the visual QC per pipeline and per cohort

First, we compared the output of visual quality control (QC) regarding the visual reads and volumetric pipelines (FSL SIENAX, FSL FIRST, FreeSurfer) between the ADC and LMC (Fig. 1) using the Kruskal–Wallis test. After excluding results that failed QC, we proceeded to scrutinize the clinical and radiological characteristics of each diagnostic group (CN, MCI, AD) comparing results between ADC and LMC cohorts. As variables were not normally distributed, we used non-parametric tests, namely Kruskal–Wallis test for continuous variables and Mann–Whitney *U* tests for categorical variables.

We then focused on the measures of GMV and HCV, each assessed with visual reads (GCA for GMV, and MTA for HCV), FSL and FreeSurfer. We used Kendall’s rank correlations to examine concordance between visual reads and quantitative volumetric measures, while concordance between FSL and FreeSurfer output was examined with Pearson’s correlation.

To establish which measure better discriminates the diagnostic groups on the base of GMV and HCV, we derived receiver operating characteristic (ROC) curves for comparisons of interest, i.e., CN vs AD, and CN vs MCI. Corresponding areas under the curve (AUCs) were compared using DeLong’s test [25] for FSL vs FreeSurfer (continuous measures), while for comparison with visual reads, we used a bootstrap test for two correlated ROC curve (continuous vs ordinal categorical measures; boot number = 2000) [26].

Significance was set at *p* value < 0.05. All statistical analyses were performed with R, version 3.6.0 (R Foundation for Statistical Computing, https://www.r-project.org/).

## Results

### Study participants

Demographics, clinical, and radiological characteristics of the study participants are reported in Table 1. Overall, patients from the LMC were older than those from the ADC independent of their diagnosis. Sex ratio was roughly equally distributed among the different groups and cohorts. The diagnostic groups were distributed as follows: 214 CN (29.2%; of which 174 ADC, 40 LMC), 279 MCI (38.1% of which 209 ADC, 70 LMC), and 205 with AD dementia (28.0% of which 109 ADC, 96 LMC). As expected, MMSE decreased progressing along the AD spectrum in both samples (*p* value < 0.001). AD patients from LMC had significantly higher MMSE scores than AD patients from ADC.

**Table 1:** Descriptive clinical and radiological characteristics of the cohorts. Data are reported as mean  ±  SD for continuous variables or *n* (%) for dichotomous variables. *p* values are reported as follows: *0.05, **0.01

	Cognitively normal (CN)			Mild cognitive impairment (MCI)			Alzheimer’s dementia (AD)		
	ADC	LMC	*p* value	ADC	LMC	*p* value	ADC	LMC	*p* value
*n*	174	40	-	209	70	-	109	96	-
Age,	61.4 ± 8.1	65.6 ± 9.9	**0.006	66.26 ± 7.7	70.4 ± 8.9	** < 0.001	67.7 ± 7.5	73.6 ± 7.5	** < 0.001
Sex, male	107 (61.5%)	21 (52.5%)	0.386	128 (61.2%)	48 (68.6%)	0.339	53 (48.6%)	41 (42.7%)	0.479
MMSE	28.1 ± 1.6	27.6 ± 5.0	0.220	26.6 ± 2.5	26.4 ± 4.3	0.654	20.5 ± 4.84	23.3 ± 5.1	** < 0.001
Visual ratings									
Fazekas	0.66 ± 0.66	1.00 ± 0.72	**0.005	1.09 ± 0.88	1.22 ± 0.74	0.269	1.00 ± 0.82	1.40 ± 0.79	** < 0.001
GCA	0.37 ± 0.54	0.57 ± 0.71	*0.048	0.71 ± 0.63	0.88 ± 0.65	*0.046	1.36 ± 0.69	0.99 ± 0.66	** < 0.001
PCA L/R avg	0.47 ± 0.62	0.88 ± 0.85	**0.001	0.71 ± 0.65	1.07 ± 0.74	** < 0.001	1.46 ± 0.77	1.40 ± 0.73	0.556
MTA L/R avg	0.33 ± 0.46	1.04 ± 0.80	** < 0.001	0.76 ± 0.83	1.33 ± 0.88	** < 0.001	1.46 ± 0.93	1.63 ± 0.82	0.163
FSL									
Total GMV [cm^3^]	764.9 ± 45.6	713.3 ± 69.6	** < 0.001	740.9 ± 55.1	672.7 ± 64.3	** < 0.001	700.6 ± 42.8	638.3 ± 64.7	** < 0.001
HCV L/R avg [cm^3^]	2.97 ± 0.54	2.66 ± 0.62	**0.004	2.70 ± 0.57	2.56 ± 0.63	0.104	2.37 ± 0.55	2.17 ± 0.51	*0.022
Scaling factor	1.28 ± 0.12	1.28 ± 0.13	0.953	1.28 ± 0.13	1.26 ± 0.12	0.185	1.31 ± 0.12	1.31 (0.13)	0.862
FreeSurfer									
Total GMV [cm^3^]	625.9 ± 36.7	565.0 ± 57.9	** < 0.001	604.8 ± 47.4	532.4 ± 52.8	** < 0.001	587.3 ± 34.4	528.1 ± 50.4	** < 0.001
HCV L/R avg [cm^3^]	3.91 ± 0.39	3.74 ± 0.62	*0.050	3.60 ± 0.49	3.32 ± 0.52	** < 0.001	3.33 ± 0.47	3.15 ± 0.49	*0.013
TIV [cm^3^]	1543.0 ± 151.5	1555.9 ± 153.2	0.658	1532.8 ± 160.1	1575.2 ± 179.9	0.082	1518.8 ± 155.1	1502.4 ± 146.1	0.462

Cerebrovascular burden, assessed with the Fazekas rating scale, was significantly higher in LMC patients in CN and AD groups, but not in MCI. GMV and HCV markers from visual ratings and volumetric pipelines showed higher degree of atrophy in the AD spectrum (*p* value < 0.001) in both settings.

**Fig. 2:**
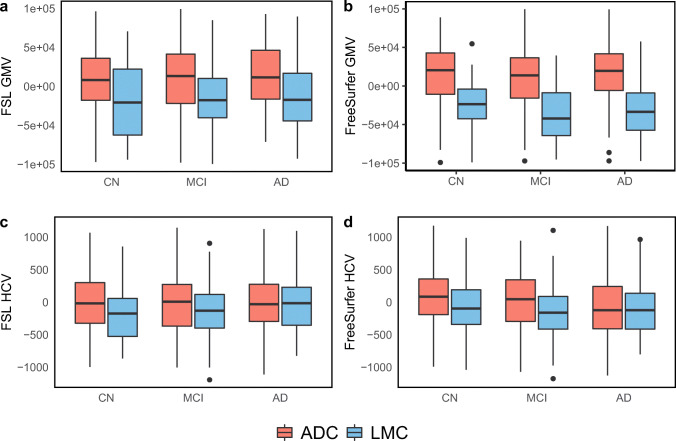
**a**–**d** Normalized HCV and GMV calculated with FSL and FreeSurfer pipelines per diagnostic group (CN, MCI, AD) and per cohort (ADC, LMC). Measures are displayed as residuals, corrected for age and sex

Quantitative volumetric values of GMV were lower in subjects from LMC compared to ADC in all groups (CN, MCI, AD) after correction for age and sex, independently of the pipeline used. The same was true for HCV measures, except in the AD groups, where no significant differences between the two different cohorts were found independent of the pipeline used (Fig. 2).

Specifically, LMC patients demonstrated significantly lower GMV and HCV values compared to ADC patients with all pipelines at all groups, *except* for PCA and MTA in dementia stage and FSL HCV at the MCI stage (Table 1).

### MRI quality control

An overview of the visual QC results is shown in Fig. 1. Trends in failure rates of each pipeline followed similar patterns in the ADC and LMC. As expected, almost all scans were suitable for visual rating (failed QC for visual reads ADC = 2.2%; LMC = 1.5%). Regarding quantification, FSL was the most failure-prone, independent of the cohort (scans failing SIENAX QC: ADC = 4.1%, LMC = 32.5%; failing FIRST QC: ADC = 6.1%, LMC = 20.9%). FreeSurfer performed better with 2.2% QC failures for ADC and 15% for LMC. For all automatic pipelines, the failure rate was significantly higher in the multicenter LMC compared to mono-center, academic ADC (*p* < 0.001). A detailed description of the failure rate per site and the scanning protocols of each site are reported in Table S1 of the Supplementary Materials. While the majority of patients from the ADC sample were scanned on a 3-T scanner, most of the patients from the LMC sample were scanned on 1.5-T scanners. Furthermore, in the LMC, failure rate seemed to follow a site-related pattern.

### Concordance between visual atrophy scores and quantitative MR metrics

**Fig. 3:**
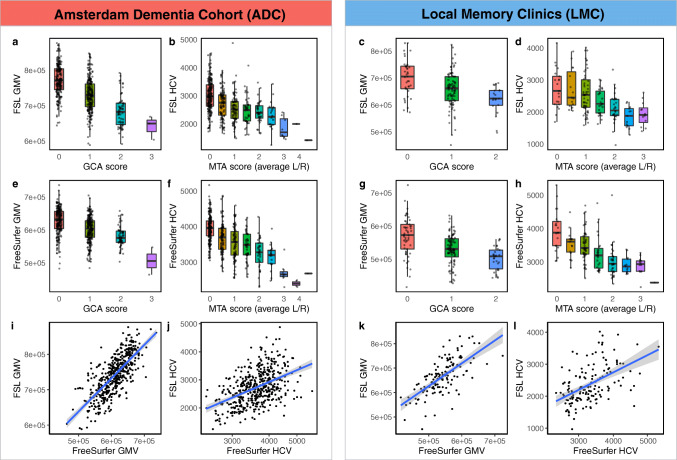
Concordance between visual reads and volumetric outcomes of GMV (**a**, **c**, **e**, **g**, **i**, **k**) and HCV (**b**, **d**, **f**, **h**, **j**, **l**). GMV was assessed through GCA visual rating, FSL SIENAX, and FreeSurfer. Similarly, HCV was assessed through MTA, FSL FIRST, and FreeSurfer. HCV were averaged between left and right (L/R) hemispheres. Volumetric outcomes (FSL and FreeSurfer) were normalized for head size and they are reported in [mm^3^]

As expected, strong correlations were found between visual ratings, FSL, and FreeSurfer outcomes of GMV and HCV respectively (Table 2, *p* value < 0.001), as shown in Fig. 3. Correlation coefficients were similar for ADC and LMC. Concordance levels were higher between visual ratings and volumetric measures after normalization for head size. On the contrary, the correlation coefficient of the volumetric output of FSL and FreeSurfer for GMV and HCV was higher before normalization, due to the different normalization procedure of the two different pipelines (Table 2).

**Table 2:**
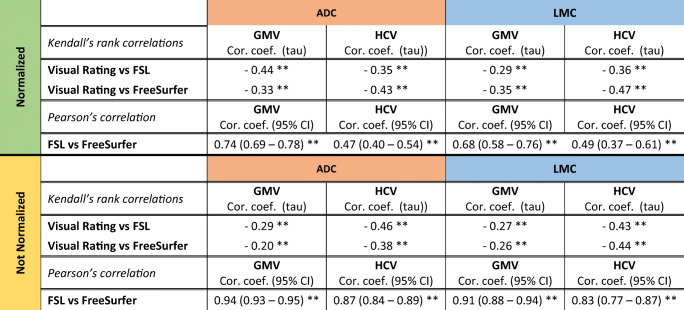
Kendall’s rank correlations between visual rating scales (GCA and MTA respectively) and volumetric measures of GMV and HCV (with FSL and FreeSurfer pipelines respectively) and Pearson’s correlation between FSL and FreeSurfer measures of GMV and HCV before (*bottom*) and after (*top*) normalization for head size. All *p* values were < 0.001 and are indicated with **

### Diagnostic performance of MRI metrics

**Fig. 4:**
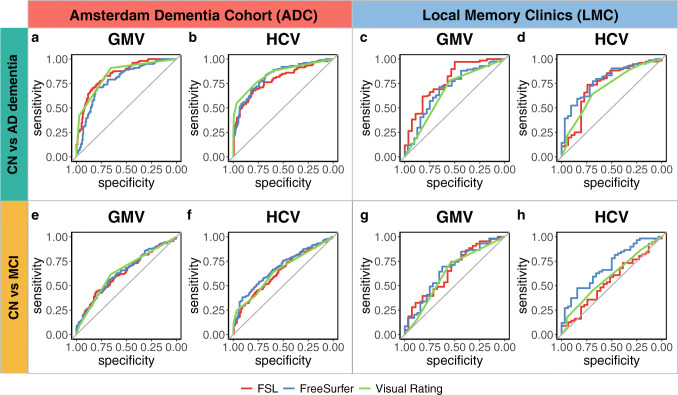
ROC curves distinguishing CN vs AD (top, **a**–**d**) and CN vs MCI (bottom, **e**–**h**) on the base of visual rating (green), FSL (red), and FreeSurfer outcomes of GMV and HCV. Results are reported separately for the Amsterdam Dementia Cohort (ADC, left panel, **a**, **b**, **e**, **f**) and local memory clinics (LMC, right panel, **c**, **d**, **g**, **h**)

ROC curves distinguishing CN vs AD and CN vs MCI on the base of GMV and HCV as assessed with visual ratings, FSL, and FreeSurfer are shown in Fig. 4. AUC of the ROC curves and results of comparisons between the different methods are reported in Table 3. In line with the expected degree of neurodegeneration per group, AUCs were higher when distinguishing CN vs AD and performance decreased for CN vs MCI.

**Table 3:**
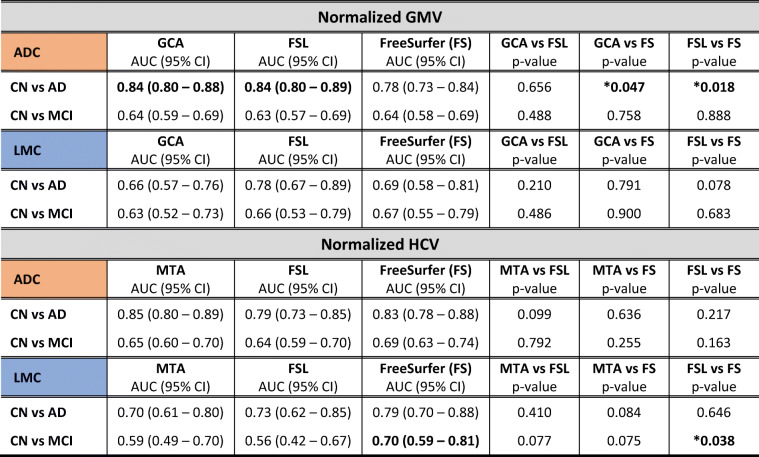
Ability of visual reads, FSL, and FreeSurfer (FS) to distinguish CN vs AD and CN vs MCI based on GMV and HCV outcomes. Area under the curve (AUC) of ROC curves is reported with 95% confidence interval. *p* values are obtained through DeLong’s method when comparing FSL vs FS and with bootstrap test for two correlated ROC curves when comparing visual reads against FSL or FS (boot number = 2000)

The discriminative power among groups was consistently higher in the ADC compared to LMC.

Within the LMC, FreeSurfer performed significantly better than FSL (*p* value = 0.038) and slightly better than MTA (*p* value = 0.077) in distinguishing CN vs MCI on the base of HCV (AUC_MTA_: 0.59, 95% CI: 0.49–0.70; AUC_FSL_ 0.55, 95% CI: 0.42–0.67; AUC_FreeSurfer_: 0.70, 95% CI: 0.59–0.81). No other significant differences in performance between visual ratings and volumetric measures were found when distinguishing clinical groups within the LMC.

Within the ADC, the best discriminative power between CN and AD was demonstrated for MTA, although AUCs for the quantitative HCV values were not significantly inferior (AUC_MTA_: 0.85, 95% CI: 0.80–0.89; AUC_FSL_ 0.79, 95% CI: 0.73–0.75; AUC_FreeSurfer_: 0.83, 95% CI: 0.78–0.88). For global atrophy, GCA visual rating scale and FSL volumes outperformed FreeSurfer (AUC_GCA_: 0.84, 95% CI: 0.80–0.88; AUC_FSL_ 0.84, 95% CI: 0.80–0.89; AUC_FreeSurfer_: 0.78, 95% CI: 0.73–0.84; *p* value GCA vs FreeSurfer: 0.047; *p* value FSL vs FreeSurfer: 0.018).

The results of the same analyses with non-normalized GMV and HCV data are reported in Table S2 of the Supplementary Materials.

## Discussion

We compared the feasibility of determining gray matter and hippocampal atrophy through semi-quantitative (using visual rating scales) and quantitative (automatic software) assessments in a real-life clinical setting of local memory clinics within The Netherlands. Automated analysis failed in up to 32% of cases without protocol optimization, much more frequent than in an academic setting. We showed that visual rating scales have a lower failure rate than quantitative analyses and have a similar discriminative power to discern clinical stages of Alzheimer’s disease.

MRI biomarkers are fundamental in the assessment of patients with Alzheimer’s disease, especially at the early stages, as indicated by the strategic roadmap for early diagnosis of Alzheimer’s disease based on biomarkers [17]. HCV, in particular, has been shown to add specificity to the diagnosis of Alzheimer’s disease, even in the early disease stages [3, 4, 17, 27]. Our results confirm that both GMV and HCV can be used in distinguishing clinical stages along the AD spectrum, and support the clinical validity of these biomarkers, and in particular visual reads results, in light of their performance in distinguishing diagnostic groups along the Alzheimer’s continuum.

Failure rate in LMC differed based on the software used; we focused on two popular freeware solution only (FSL and FreeSurfer) and did not examine commercial software packages. In those subjects where quantification was successful, quantification did not lead to higher accuracy than visual rating by an experienced neuroradiologist. Based on our findings, the diagnostic performance of visual rating scales from an experienced reader is sufficient and generally comparable to that of volumetric outcomes, with the additional advantage of suffering less from quality issues in the images, even in non-academic settings. A possible advantage of quantification using automated pipelines is that they provide a greater level of detail, being continuous variables. Additional advantages of the use of quantitative outcomes could be to expedite the radiological assessment of MRI scans and decrease subjectivity if well integrated in the radiological flow. This could become more relevant with the continuous improvement of the segmentation techniques and the advent of artificial intelligence and automatic decision support tools that can lead to more precise volumetric measures [28–30]. On the other hand, issues related to training and technical expertise required to produce such volumetric outputs currently prevent a practical implantation in the clinics.

The images we used for volumetric quantifications came from real-life clinical settings, and were thus variable in terms of scanners, acquisition protocol parameters, and general quality. Our results suggest that the quality of ADC MRI scans was generally higher when compared to LMC. This might partially reflect efforts to achieve protocol standardization across scanners within the ADC [19] and different levels of experience between academic and non-academic centers. Moreover, most of the data from the ADC as acquired with 3T scanners, as opposed to the LMC where most data were collected on 1.5-T scanners. This has probably impacted the number of failures and the quality of the segmentations in favor of the ADC. We reported scanning protocol details in the Supplementary Materials. Although the disentangling of technical scanning parameters that could affect volumetric measurement with automatic software goes beyond the scope of this study, research in this direction would certainly aid in the translation of automatic software use in the clinical practice. Finally, data collection also differed between the academic and non-academic centers, as the LMC sample was prospectively collected, while the ADC sample was retrospectively included in the analyses as the data were already available.

Moreover, the two samples had different clinical characteristics, as patients referred to academic centers are usually clinically more challenging, while older patients with a less complex diagnostic profile were investigated at LMC. This is confirmed by the significant differences between the ADC and LMC samples in age, MMSE score, vascular burden, and respective numbers of diagnostic groups, as patients from LMC generally presented in more advanced stages of disease (MCI, dementia), although MMSE values within the dementia stage were higher in the LMC group, suggesting that this screening test does not capture clinical nuances. In line with this hypothesis, the GMV and HCV were consistently lower in individuals from LMC in all syndromic groups, independently of the pipeline used. This might have also influenced the diagnostic performance within LMC, as the volumetric assessment of atrophic brains is more challenging, due to increased segmentation uncertainty as a function of the ratio between the surface area and the volume of the structure [31].

The use of real-life clinical data both from academic and non-academic memory clinics is a strength of this study, making results applicable to a clinical setting. Although follow-up data were not available for this study, it has been previously demonstrated that volumetric measures are more sensitive to change than visual reads. On the other hand, these measures are also susceptible to changes as a consequence of variations in scanning protocols and technical parameters, which might also be a pitfall. A limitation of this study is that we did not study visual reads by local (neuro)radiologist; likewise, the volumetric quantification and QC were performed centrally. This is also a strength, as variability was limited, making our results more robust. We only used two popular freeware solutions (FSL and FreeSurfer) for quantification of GMV and HCV. Although a comprehensive comparison of all possible methods for GMV and HCV quantification methods and the investigation of their technical peculiarities was beyond the scope of this study, these methodological differences, coupled with our consequent choices (for instance in normalization for head size), might have introduced a bias in the results, as no consensus exists regarding the correct way to analyze volumetric data. Finally, results might have improved with quality assessment of intermediate analysis steps. Nevertheless, we aimed at reproducing as much as possible what could happen in a real-life clinical setting where time and resources limit the feasibility of step-by-step QC of standardized pipelines.

In conclusion, our results indicate that brain MRI scans from non-academic memory clinics have a considerable failure rate for the quantification of GMV and HCV without protocol optimization. Quantitative volumetric outputs of automatized software were generally not superior to visual ratings by an experienced radiologist, suggesting that, given the time constraints and limited resources of real-life clinical settings, the use of such software may not yet be ready for use in the radiological workup of individuals with suspected Alzheimer’s disease. Although their implementation in the clinical world remains still complex, quantitative measures remain promising tools to standardize the ratings, save time to manual operators, and give more precise quantifications of brain atrophy.

## Supplementary Information

Below is the link to the electronic supplementary material.

Supplementary file1 (DOCX 36 KB)

## Abbreviations

AD: Alzheimer’s dementia
ADC: Amsterdam Dementia Cohort
AUC: Area under the curve
CN: Cognitively normal
GCA: Global cortical atrophy
GMV: Gray matter volume
HCV: Hippocampal volume
LMC: Local memory clinics
MCI: Mild cognitive impairment
MMSE: Mini-Mental State Examination
MRI: Magnetic resonance imaging
MTA: Medial temporal atrophy
PCA: Posterior cortical atrophy
QC: Quality check
ROC: Receiver operating characteristic

## Declarations

### Guarantor

The scientific guarantor of this publication is Prof. Dr. Frederik Barkhof.

### Statistics and biometry

No complex statistical methods were necessary for this paper.

### Study subjects or cohorts overlap

Some study subjects of ADC have been previously reported in van der Flier WM, Scheltens P (2018) Amsterdam Dementia Cohort: performing research to optimize care. *J. Alzheimers Dis*. 62:1091–1111.

### Methodology

• This is a multicentric cross-sectional study

• Data from LMC were collected prospectively

• Data from ADC were collected retrospectively.
