# Supplementary material for: Clinical applicability of quantitative atrophy measures on MRI in patients suspected of Alzheimer’s disease
Source: Eur Radiol. 2022 May 31;32(11):7789–99. doi: 10.1007/s00330-021-08503-7 (PMC9668763; doi:10.1007/s00330-021-08503-7)
Supplement: Supplementary file 1 — Supplementary file1 (DOCX 36 KB) [file 330_2021_8503_MOESM1_ESM.docx]

**Supplementary Materials**

**Table S1. Scanning protocols and failed analyses report per site.** The failure rates are reported in numbers and percentage over the total number of acquired scans per cohort (ADC, n = 492 and LMC, n = 206).

*Acronyms*: COR, coronal; FFE, fast field echo; FSPGR, fast spoiled gradient echo; MPRAGE, magnetization prepared rapid acquisition gradient echo; TFE, turbo field echo; SAG, sagittal. *Sites LMC*: CA, NoordWest Ziekenhuis Groep, Alkmaar; CL, Medisch Centrum Leeuwarden, Leeuwarden; CZ, Catharina Hospital, Eindhoven; EZ, Elisabeth-TweeSteden Ziekenhuis, Tilburg; JB, Jeroen Bosch Hospital, 's-Hertogenbosch; KG, Spaarne Gasthuis, Haarlem; LA, Medisch Centrum Leeuwarden, Leeuwarden; TE, Tergooi Hospital, Blaricum.

| **Site** | **Manufacturer** | **Model** | **Field strength** | **Series description** | **Pixel size (mm^2^)** | **Slice thickness** | **N** | **Failed QC**  **FSL SIENAX** | **Failed QC**  **FSL FIRST** | **Failed QC**  **FreeSurfer** |  |
| --- | --- | --- | --- | --- | --- | --- | --- | --- | --- | --- | --- |
| **ADC** | | | | | | | | | | | |
|  | Siemens | Avanto | 1.5 T | MPRAGE COR | 1.00 × 1.00 | 1.5 mm | 11 | 3 (0.6%) | 0 (0.0%) | 0 (0.0%) |  |
|  | Siemens | Sonata | 1.5 T | MPRAGE COR | 1.00 × 1.00 | 1.5 mm | 34 | 1 (0.2%) | 0 (0.0%) | 0 (0.0%) |  |
|  | Siemens | Vision | 1.5 T | MPRAGE COR | 0.98 × 1.98 | 1.5 mm | 2 | 1 (0.2%) | 0 (0.0%) | 0 (0.0%) |  |
|  | GE | SignaHDxt | 1.5 T | FSPGR SAG | 0.98 × 1.98 | 1.5 mm | 18 | 0 (0.0%) | 0 (0.0%) | 0 (0.0%) |  |
|  | GE | SignaHDxt | 3 T | FSPGR SAG | 0.98 × 1.98 | 1.5 mm | 418 | 15 (3.1%) | 30 (6.1) | 10 (2.0%) |  |
|  | Philips | Ingenuity PET/MR | 3 T | TFE SAG | 1.00 × 1.00 | 1.0 mm | 7 | 0 (0.0%) | 0 (0.0%) | 1 (0.2%) |  |
|  | Toshiba | Titan | 3 T | FFE SAG | 1.00 × 1.00 | 1.0 mm | 2 | 0 (0.0%) | 0 (0.0%) | 0 (0.0%) |  |
| **Total** | - | - | - | - | - | - | 492 | 20 (4.1%) | 30 (6.1%) | (2.2%) |  |

| **Site** | **Manufacturer** | **Model** | **Field strength** | **Series description** | **Pixel size (mm^2^)** | **Slice thickness** | **N** | **Failed QC**  **FSL SIENAX** | **Failed QC**  **FSL FIRST** | **Failed QC**  **FreeSurfer** |
| --- | --- | --- | --- | --- | --- | --- | --- | --- | --- | --- |
| **LMC** | | | | | | | | | | |
| **CA** | Siemens | Avanto | 1.5 T | T1 MPR COR | 0.98 × 0.98 | 1 mm |  |  |  |  |
|  | Siemens | Avanto | 1.5 T | T1 MPR COR | 0.49 × 0.49 | 1 mm |  |  |  |  |
|  | Siemens | Espree | 1.5 T | T1 MPR COR | 0.98 × 0.98 | 1 mm |  |  |  |  |
|  | Siemens | Espree | 1.5 T | T1 MPR COR | 0.49 × 0.49 | 1 mm |  |  |  |  |
|  | Siemens | Symphony | 1.5 T | T1 3D MPR COR | 0.51 × 0.51 | 1.1 mm |  |  |  |  |
|  | Siemens | Symphony | 1.5 T | T1 3D MPR COR | 1.17 × 1.17 | 1.2 mm |  |  |  |  |
|  | Siemens | Symphony | 1.5 T | T1 3D MPR COR | 0.86 × 0.86 | 3 mm |  |  |  |  |
| **Subtotal** | - | - | - | - | - | - | 50 | 13 (6.3%) | 11 (5.3%) | 2 (1.0%) |
| **CL** | Philips | Achieva | 1.5 T | T1W 3D FFE | 0.90 × 0.90 | 1.6 mm |  |  |  |  |
|  | Philips | Achieva | 1.5 T | T1W 3D TFE | 1.00 × 1.00 | 1.2 mm |  |  |  |  |
|  | Philips | Achieva | 3 T | T1W 3D TFE | 0.94 × 0.94 | 1 mm |  |  |  |  |
|  | Philips | Intera | 1.5 T | T1W 3D TFE | 1.00 × 1.00 | 1.2 mm |  |  |  |  |
|  | Philips | Intera | 1.5 T | T1W 3D TFE SAG | 1.00 × 1.00 | 3 mm |  |  |  |  |
|  | Philips | Intera | 1.5 T | T1W 3D TFE COR | 1.00 × 1.00 | 3 mm |  |  |  |  |
| **Subtotal** | - | - | - | - | - | - | 23 | 3 (1.5%) | 4 (1.9%) | 3 (1.5%) |
| **CZ** | Philips | Ingenia | 1.5 T | T1W 3D TFE | 0.90 × 0.90 | 2.8 mm | 9 | 8 (3.8%) | 2 (1.0%) | 8 (3.8%) |
| **EZ** | Philips | Achieva | 3 T | T1W 3D TFE | 1.00 × 1.00 | 1 mm |  |  |  |  |
|  | Philips | Intera | 1.5 T | T1w 3d FFE | 0.80 × 0.80 | 1.6 mm |  |  |  |  |
| **Subtotal** | - | - | - | - | - | - | 26 | 7 (3.4%) | 2 (1.0%) | 2 (1.0%) |
| **JB** | Siemens | Espree | 1.5 T | COR T1 TIR | 0.45 × 0.45 | 4 mm | 21 | 2 (1.0%) | 0 (0.0%) | 1 (0.5%) |
| **KG** | Philips | Achieva | 1.5 T | 3D T1 FFE | 0.47 × 0.47 | 2 mm |  |  |  |  |
|  | Philips | Achieva | 1.5 T | 3D T1 FFE | 0.47 × 0.47 | 2 mm |  |  |  |  |
|  | Philips | Achieva dStream | 1.5 T | 3D T1 TFE | 0.57 × 0.57 | 2 mm |  |  |  |  |
|  | Philips | Achieva dStream | 1.5 T | MPR sag test | 0.57 × 0.57 | 2 mm |  |  |  |  |
|  | Philips | Achieva dStream | 1.5 T | 3D T1 FFE sag | 0.63 × 0.63 | 1.4 mm |  |  |  |  |
| **Subtotal** | - | - | - | - | - | - | 68 | 27 (13.1%) | 17 (8.3%) | 9 (4.3%) |
| **LA** | Siemens | Avanto | 1.5 T | T1 MPR SAG P2 ISO | 0.98 × 0.98 | 1 mm |  |  |  |  |
|  | GE | Signa HDxt | 1.5 T | SAG T1 3D FSPGR | 0.47 × 0.47 | 1.6 mm |  |  |  |  |
|  | GE | Signa HDxt | 1.5 T | COR 2 mm | 0.47 × 0.47 | 2 mm |  |  |  |  |
| **Subtotal** | - | - | - | - | - | - | 3 | 2 (1.0%) | 2 (1.0%) | 2 (1.0%) |
| **TE** | Siemens | Aera | 1.5 T | T1 FL3D SAG ISO | 0.98 × 0.98 | 1 mm |  |  |  |  |
|  | Siemens | Aera | 1.5 T | T1 TIR COR | 0.72 × 0.72 | 4 mm |  |  |  |  |
|  | Siemens | Avanto | 1.5 T | T1 TIR COR | 0.45 × 0.45 | 2 mm |  |  |  |  |
| **Subtotal** | - | - | - | - | - | - | 6 | 5 (2.4%) | 5 (2.4%) | 4 (1.9%) |
| **Total** | - | - | - | - | - | - | 206 | 67 (32.5%) | 43 (20.9) | 31 (15.0%) |

**Table S2.** Ability of visual reads, FSL, and FreeSurfer (FS) to distinguish CN vs AD and CN vs MCI based on non-normalized GMV and HCV outcomes. Area under the curve (AUC) of ROC curves is reported with 95% confidence interval. *P-values* are obtained through DeLong method when comparing FSL vs FS and with bootstrap test for two correlated ROC curves when comparing visual reads against FSL or FS (boot number = 2000).

| **Non-Normalised GMV** | | | | | | |
| --- | --- | --- | --- | --- | --- | --- |
| **ADC** | **GCA**  AUC (95% CI) | **FSL**  AUC (95% CI) | **FreeSurfer (FS)**  AUC (95% CI) | **GCA vs FSL**  p-value | **GCA vs FS** p-value | **FSL vs FS**  p-value |
| **CN vs AD** | **0.84 (0.80 – 0.88)** | 0.78 (0.72 – 0.83) | 0.71 (0.65 – 0.77) | ***0.014** | ***<0.001** | ***<0.001** |
| **CN vs MCI** | 0.64 (0.59 – 0.69) | 0.61 (0.55 – 0.67) | 0.61 (0.56 – 0.67) | 0.342 | 0.464 | 0.955 |
| **LMC** | **GCA**  AUC (95% CI) | **FSL**  AUC (95% CI) | **FreeSurfer (FS)**  AUC (95% CI) | **GCA vs FSL**  p-value | **GCA vs FS** p-value | **FSL vs FS**  p-value |
| **CN vs AD** | 0.66 (0.57 – 0.76) | **0.80 (0.70 – 0.89)** | 0.75 (0.65 – 0.85) | 0.139 | 0.220 | ***0.006** |
| **CN vs MCI** | 0.63 (0.52 – 0.73) | 0.61 (0.47 – 0.74) | 0.62 (0.49 – 0.74) | 0.145 | 0.397 | 0.346 |
| **Non-Normalised HCV** | | | | | | |
| **ADC** | **MTA**  AUC (95% CI) | **FSL**  AUC (95% CI) | **FreeSurfer (FS)**  AUC (95% CI) | **MTA vs FSL**  p-value | **MTA vs FS** p-value | **FSL vs FS**  p-value |
| **CN vs AD** | 0.85 (0.80 – 0.89) | 0.83 (0.78 – 0.89) | 0.82 (0.76 – 0.87) | 0.714 | 0.424 | 0.821 |
| **CN vs MCI** | 0.65 (0.60 – 0.70) | 0.69 (0.64 – 0.74) | 0.68 (0.63 – 0.73) | 0.208 | 0.165 | 0.934 |
| **LMC** | **MTA**  AUC (95% CI) | **FSL**  AUC (95% CI) | **FreeSurfer (FS)**  AUC (95% CI) | **MTA vs FSL**  p-value | **MTA vs FS** p-value | **FSL vs FS**  p-value |
| **CN vs AD** | 0.70 (0.61 – 0.80) | 0.76 (0.66 – 0.87) | **0.83 (0.75 -0.92)** | 0.182 | ***0.007** | 0.336 |
| **CN vs MCI** | 0.59 (0.49 – 0.70) | 0.60 (0.47 – 0.72) | 0.69 (0.57 – 0.81) | 0.549 | 0.147 | 0.098 |
